# Xiaoaiping injection combined with chemotherapy for advanced gastric cancer: An updated systematic review and meta-analysis

**DOI:** 10.3389/fphar.2022.1023314

**Published:** 2022-09-30

**Authors:** Xing Qin Zhou, Ya Zhou Chang, Chao Yan Shen, Jie Han, Ren An Chang

**Affiliations:** ^1^ Department of Radiotherapy, Affiliated Hospital of Nantong University, Nantong, China; ^2^ School of Medicine, Southeast University, Nanjing, China

**Keywords:** Xiaoaiping injection, advanced gastric cancer, meta-analysis, systemic review, updated

## Abstract

**Aim:** To evaluate the clinical efficacy and safety of Xiaoaiping injection combined with chemotherapy in the treatment of advanced gastric cancer by meta-analysis.

**Methods:** Seven databases, including China National Knowledge Infrastructure (CNKI), Wanfang Database, VIP Database, Cochrane Library, PubMed, Embase, and Web of Science, were searched by computer for randomized controlled clinical trials of Xiaoaiping injection combined with chemotherapy in the treatment of gastric cancer. Risk of bias assessment and meta-analysis were performed by Review Manager 5.3 software.

**Results:** There were 16 articles that met the inclusion criteria, with a total of 1,236 patients, 617 in the observation group and 619 in the control group. The results of meta-analysis showed that the observation group was better than chemotherapy alone control group in RR [OR = 1.86, *p* < 0.00001]; disease control rate (DCR) [OR = 2.45, *p* < 0.00001]; Karnofsky performance status (KPS) score [OR = 3.21, *p* < 0.00001] or [MD = 7.73, *p* = 0.001]. In terms of biochemical indicators, Xiaoaiping significantly reduced inflammation factors level, including tumor necrosis factor alpha (TNF-α) [MD = −15.00, *p* < 0.00001]; interleukin-6 (IL-6) [MD = −13.00, *p* < 0.00001]; C-reaction protein (CRP) [MD = −5.80, *p* < 0.00001]. Xiaoaiping could enhance immune function, significantly reducing myeloid-derived suppressor cells (MDSCs) [MD = −6.20, *p* < 0.00001] and Treg [MD = −1.70, *p* < 0.00001]. Xiaoaiping injection combined with chemotherapy could significantly decrease tumor markers, including carcinoembryonic antigen (CEA) [MD = −11.64, *p* < 0.00001]; CA199 [MD = −33.57, *p* = 0.02]; CA242 [MD = −20.66, *p* < 0.00001]; CA125 [MD = −12.50, *p* = 0.0005]. In the comparison of adverse reactions, the incidence rate of Xiaoaiping injection group was significantly lower than that of control group. The funnel plot showed that the left and right sides are basically symmetrical, and it can be considered that there is no obvious publication bias.

**Conclusion:** Xiaoaiping injection combined with chemotherapy has better curative effect and less adverse reactions in the treatment of gastric cancer. However, limited by the quality of the included studies, more high-quality studies are still needed to be verified.

**Systematic Review Registration**: [https://www.crd.york.ac.uk/prospero/display_record.php?ID=CRD42022353842], identifier [CRD42022353842].

## Introduction

Gastric cancer is a common malignant tumor of digestive tract, and its morbidity and mortality are high among malignant tumors, which seriously affects the quality of life and physical health of patients (Parkin et al., 1999; Chen et al., 2016). Clinically, it is more common in patients with advanced gastric cancer, which basically loses the technique of radical surgery, and needs to be intervened by anti-tumor drugs such as radiotherapy and chemotherapy to prolong the life cycle and improve the quality of life (Smyth et al., 2020; Thrift and El-Serag 2020; Sung et al., 2021). Clinical strategies for the treatment of gastric cancer include XELOX (oxaliplatin + xeloda), FOLFOX6 regimen (oxaliplatin + leucovorin + 5-fluorouracil), SOX (oxaliplatin + seggio), etc. Patients often cannot tolerate the adverse reactions of radiotherapy and chemotherapy due to low immune function (Johnston and Beckman 2019). Chinese patent medicine may have the effect of increasing the efficacy of radiotherapy and chemotherapy and reducing adverse reactions. In recent years, a variety of Chinese patent medicine injections combined with chemotherapy have shown good efficacy and less side effects in the treatment of gastric cancer (Liu et al., 2015). Xiaoaiping injection is a Chinese patent medicine preparation made of *Marsdenia tenacissima* (Roxb.) Wight et Arn extract. It has the functions of clearing away heat and detoxifying, resolving phlegm and softening the pain (Chen and Liu 2004; Chen et al., 2021). It had pharmacological effects such as anti-tumor, antihypertensive and antiasthmatic, and immune regulation, clinically used for gastric cancer, lung cancer, esophageal cancer, liver cancer, and other diseases (Chen and Liu 2004). Modern pharmacological research showed the mechanisms of anti-tumor were related with promoting tumor cell apoptosis, inhibiting tumor cell proliferation and tumor blood vessel growth, and regulating immunity, etc. In this study, combined with clinical practice, the conventional chemotherapy + Xiaoaiping injection was used as the observation group, and the conventional chemotherapy was used as the control group. The application of Xiaoaiping injection provides references and suggestions.

## Materials and methods

The protocol for this review and meta-analysis has been registered on the International Prospective Register of Systematic Reviews (PROSPERO) with the registration number CRD42022353842.

### Data source

The research object is the randomized controlled trials (RCTs) of Xiaoaiping injection in the treatment of gastric cancer published at home and abroad. Seven databases were searched, including China National Knowledge Infrastructure (CNKI), Wanfang, VIP, Cochrane Library, PubMed, Embase and Web of Science. The search period is from inception until July 2022. The search terms included (Gastric Cancer) AND (Xiaoaiping Injection or Xiaoaiping).

### Inclusion criteria

1) RCT; 2) Subjects: gastric cancer confirmed by histopathological examination and other imaging data; 3) Group: observation group and control group; 4) Intervention measures: The observation group was treated with Xiaoaiping injection combined with chemotherapy for gastric cancer, and the control group was treated with chemotherapy alone, with unlimited dose and course of treatment; 5) Outcome indicators: the efficacy rate must be included.

### Exclusion criteria

1) Non-RCTs; 2) duplicate publications, conference papers, dissertations, etc.; 3) no control group; 4) animal experiments; 5) interventions that are not Xiaoaiping injections, but other formulation type, such as tablets, capsules, etc.; 6) Literature for non-gastric cancer patients.

### Literature screening and extraction

Two experienced researchers independently read and screened the literature according to the inclusion and exclusion criteria and extracted data from the final included literature. The bias risk of the included studies was evaluated according to the Cochrane Handbook’s Bias Risk Assessment Tool for RCTs. In case of disagreement, consensus was reached with the help of a third author, who comprehensively analyzed and guided whether to include or not, and finally determined the included literature, read the full text of the included literature in detail, and extracted information such as the authors, allocation methods, intervention measures, and outcome indicators.

### Statistical methods

Review Manager 5.3 software was used for statistical analysis and bias risk assessment. There are four evaluation criteria for the efficacy of drug: complete remission (CR), partial remission (PR), stable disease (SD), and progressive disease (PD). The remission rate (RR) was calculated as CR + PR, and the disease control rate (DCR) was calculated as CR + PR + SD. When performing a meta-analysis, the heterogeneity test was first carried out. *p* > 0.05 and I2 < 50% indicated the favorable homogeneity, so the fixed effect model was used to analyze. *p* < 0.05 and I2 > 50% indicated the poor homogeneity, so the random effects model was used for analysis. The count data were analyzed by odds ratio (OR); the measurement data were evaluated by mean difference (MD), and each response was expressed with 95% confidence interval (CI), and a forest plot was drawn. Finally, funnel plots were drawn to objectively and quantitatively assess the publication bias of the studies.

## Results

### General information and bias risk assessment

**FIGURE 1:**
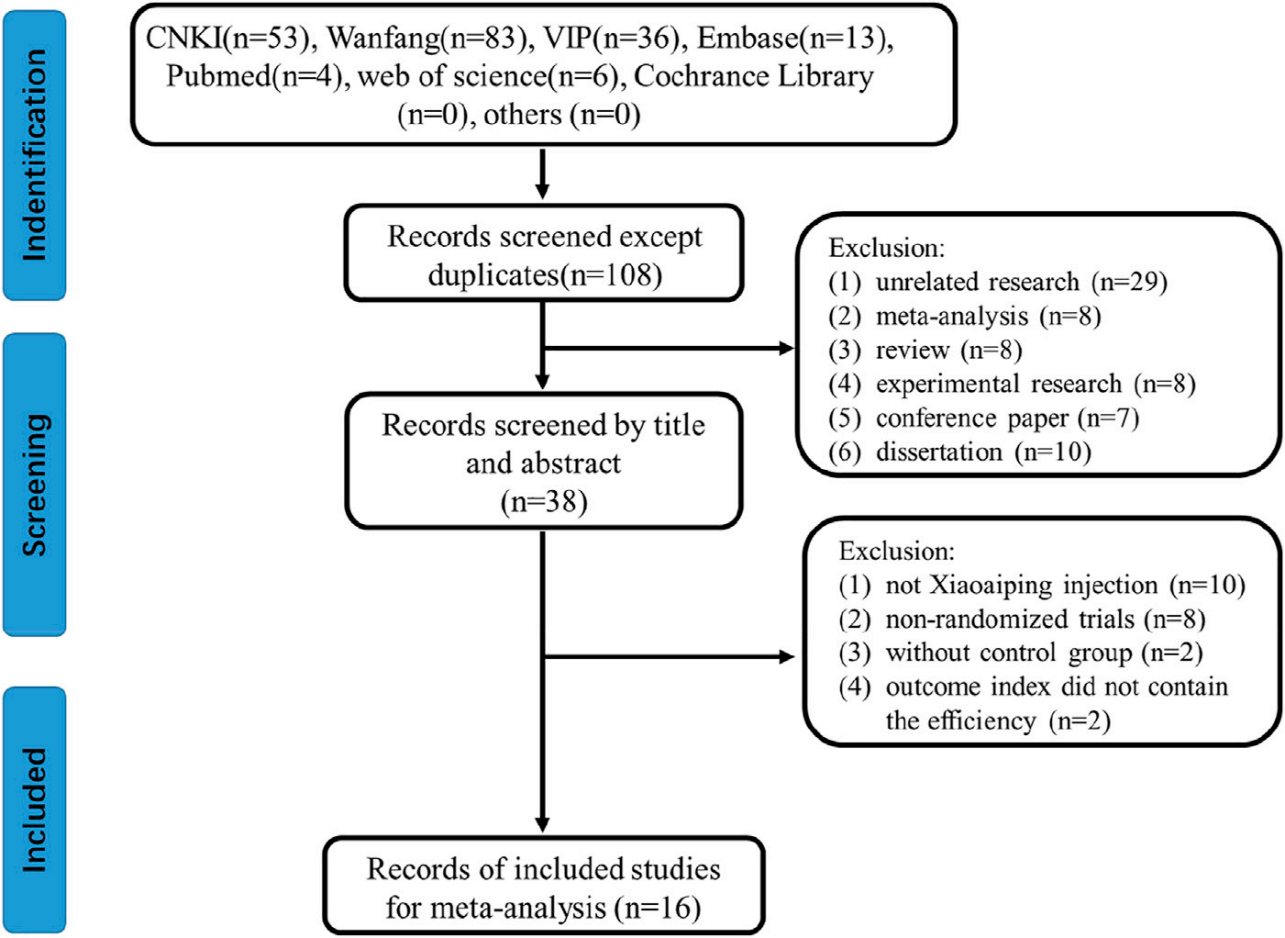
PRISMA flow diagram of this study.

**FIGURE 2:**
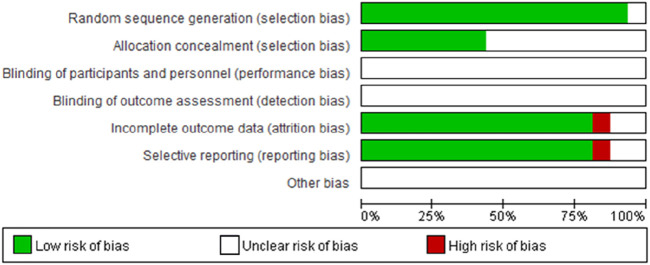
Risk-of-bias summary.

16 RCTs were finally included through computer searching, manual screening, and full text reading. The literature screening process is shown in Figure 1. A total of 1,236 patients were included in the 16 RCTs, including 617 cases in the observation group and 619 cases in the control group. Basic characteristics of the included literature were shown in Table 1. The overall bias risk assessment was shown in Figure 2.

**TABLE 1:** Baseline information of included studies.

Included trials	Allocation method	Gastric cancer staging	Cases		Interventions		XAPI dosage	Chemotherapy time	Outcomes
			Observation group	Control group	Observation group	Control group			
Lin et al. (2015)	Random	Middle-advanced	28	28	XELOX + XAPI	XELOX	60 ml/d	3 weeks as 1 course, 2 courses	RR, DCR, KPS, survival rate, adverse reactions
Guo et al. (2018)	Random number table	Middle-advanced	61	61	XELOX + XAPI	XELOX	40 ml/d	3 weeks as 1 course, 2 courses	RR, DCR, KPS, TNF-α, peripheral T lymphocyte subset levels (CD3^+^, CD4^+^, CD8^+^)
Huo and Chen (2009)	Random	Advanced	31	31	FOIFOX + XAPI	FOIFOX	unclear	4 weeks as 1 course, 2 courses	RR, KPS, adverse reactions
Wang and Chen (2018)	Random number table	Middle-advanced	40	40	XELOX + XAPI	XELOX	40 ml/d	3 weeks as 1 course, 2 courses	RR, DCR, survival rate, adverse reactions, IL-6, TNF-α, CRP, peripheral blood myeloid-derived suppressor cells (MDSCs) and regulatory T cells (Treg)
Saifuding et al. (2012)	Random number table	Advanced	33	35	FOIFOX + XAPI	FOIFOX	60 ml/d	8 weeks as 1 course, 2 courses	RR, DCR, KPS, adverse reactions, tumor markers (CEA, CA199, CA724)
Ma (2015)	Random	Advanced	23	23	SOX + XAPI	SOX	60 ml/d	3 weeks as 1 course, 2 courses	RR, KPS, adverse reactions, tumor markers (CEA, CA199, CA724)
Ruan et al. (2021)	Random number table	Advanced	42	42	SOX + XAPI	SOX	100 ml/d	3 weeks as 1 course, 2 courses	RR, DCR, KPS, adverse reactions, immune function
Deng et al. (2016)	Random	Advanced	15	15	TP + XAPI	TP	80 ml/d	3 weeks as 1 course, 2 courses	RR, DCR, KPS, adverse reactions
Zhang and Li (2015)	Random	Middle-advanced	23	25	XELOX + XAPI	XELOX	40 ml/d	3 weeks as 1 course, 2 courses	RR, DCR, KPS, adverse reactions
Gao et al. (2015)	Parity random	Advanced	92	91	XELOX + XAPI	XELOX	40 ml/d	3 weeks as 1 course, 4 courses	RR, DCR, survival rate, adverse reactions
Zheng et al. (2017b)	Random number table	Advanced	40	41	Docetaxel + Oxaliplatin + XAPI	Docetaxel + Oxaliplatin	40–60 ml/d	3 weeks as 1 course, 2–6 courses	RR, DCR, KPS, survival rate, adverse reactions
Liu and Zhu (2012)	Random	Advanced	28	28	FOIFOX + XAPI	FOIFOX	80 ml/d	2 weeks as 1 course, 4 courses	RR, DCR, KPS, adverse reactions
Ge et al. (2019)	Random number table	Advanced	40	40	Seggio Capsules + XAPI	Seggio Capsules	80 ml/d	6 weeks as 1 course, 4 courses	RR, DCR, KPS, survival rate, adverse reactions, Tumor markers (CEA, CA19-9, CA125)
Xiong et al. (2015)	Random	Advanced	32	32	SOX + XAPI	SOX	80 ml/d	3 weeks as 1 course, 4 courses	RR, DCR, survival rate, adverse reactions, TNF-α
Yan et al. (2018)	Random	Middle-advanced	29	27	XELOX + XAPI	XELOX	60 ml/d	3 weeks as 1 course	RR, DCR, survival rate, adverse reactions
Li and Ran (2016)	Random	Advanced	60	60	Irinotecan + XAPI	Irinotecan	80 ml/d	2 weeks as 1 course, 4 courses	RR, DCR, survival rate, adverse reactions, Tumor markers (CEA, CA199, CA242)

Note: XAPI, Xiaoaiping injection; XELOX, xeloda + oxaliplatin; FOIFOX, oxaliplatin + leucovorin + 5-fluorouracil; SOX, oxaliplatin + seggio capsules; TP, paclitaxel + cisplatin; KPS, Karnofsky performance status; TNF-α, tumor necrosis factor alpha; IL-6, interleukin-6; CRP, C-reaction protein; CEA/CA, carcinoembryonic antigen; MDSCs, myeloid-derived suppressor cells; Treg, regulatory T cells.

## Clinical efficacy

### Efficacy comparison

**FIGURE 3:**
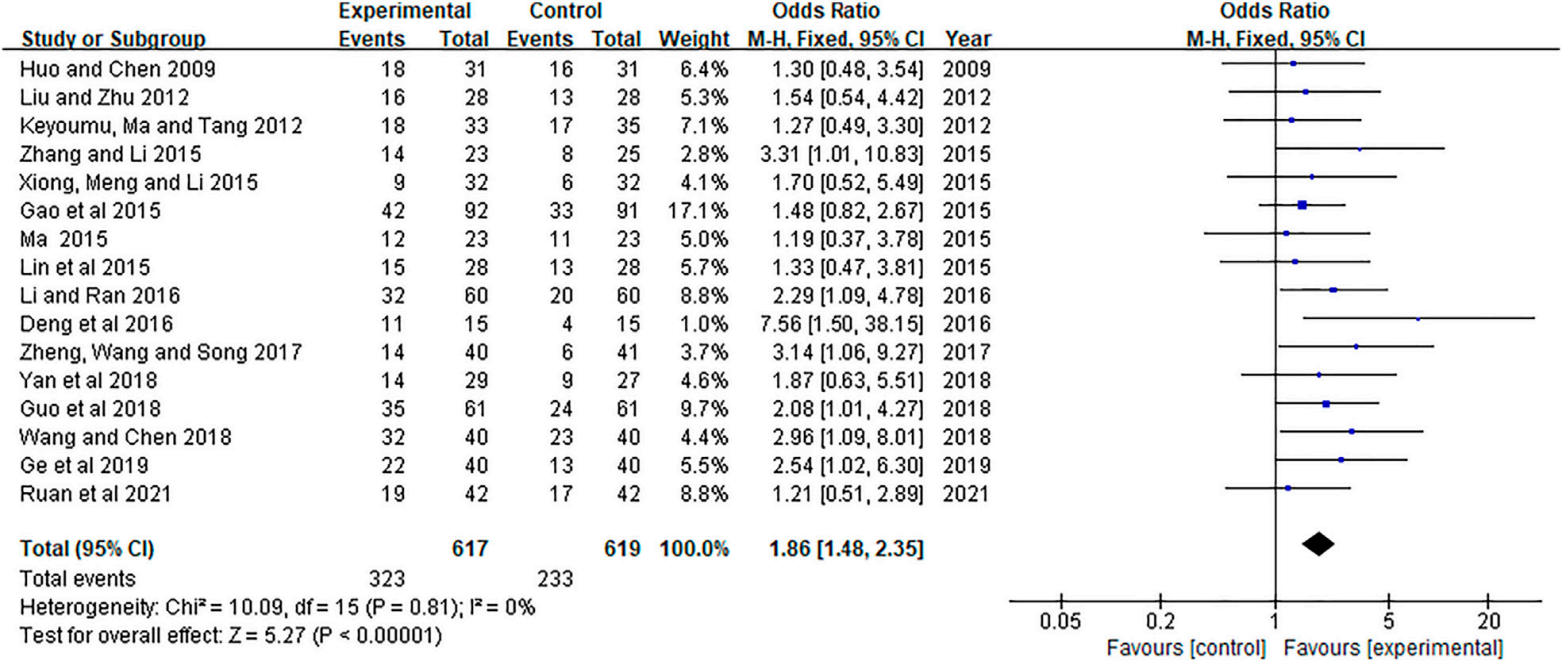
Meta-analysis of RR.

16 RCTs (Huo and Chen 2009; Liu and Zhu 2012; Saifuding et al., 2012; Gao et al., 2015; Lin et al., 2015; Ma 2015; Xiong et al., 2015; Zhang and Li 2015; Deng et al., 2016; Li and Ran 2016; Zheng et al., 2017b; Guo et al., 2018; Wang and Chen 2018; Yan et al., 2018; Ge et al., 2019; Ruan et al., 2021) included 1,236 patients reported RR (RR = CR + PR). The results were shown in Figure 3. Heterogeneity test showed *p* = 0.81, *I* ^*2*^ = 0.0%. Therefore, the fixed-effects model was used for analysis. The meta-analysis results showed that RR of the experimental group was significantly higher than that of the control group [OR = 1.86, 95% CI (1.48, 2.35), Z = 5.27, *p* < 0.00001].

### Comparison of DCR

**FIGURE 4:**
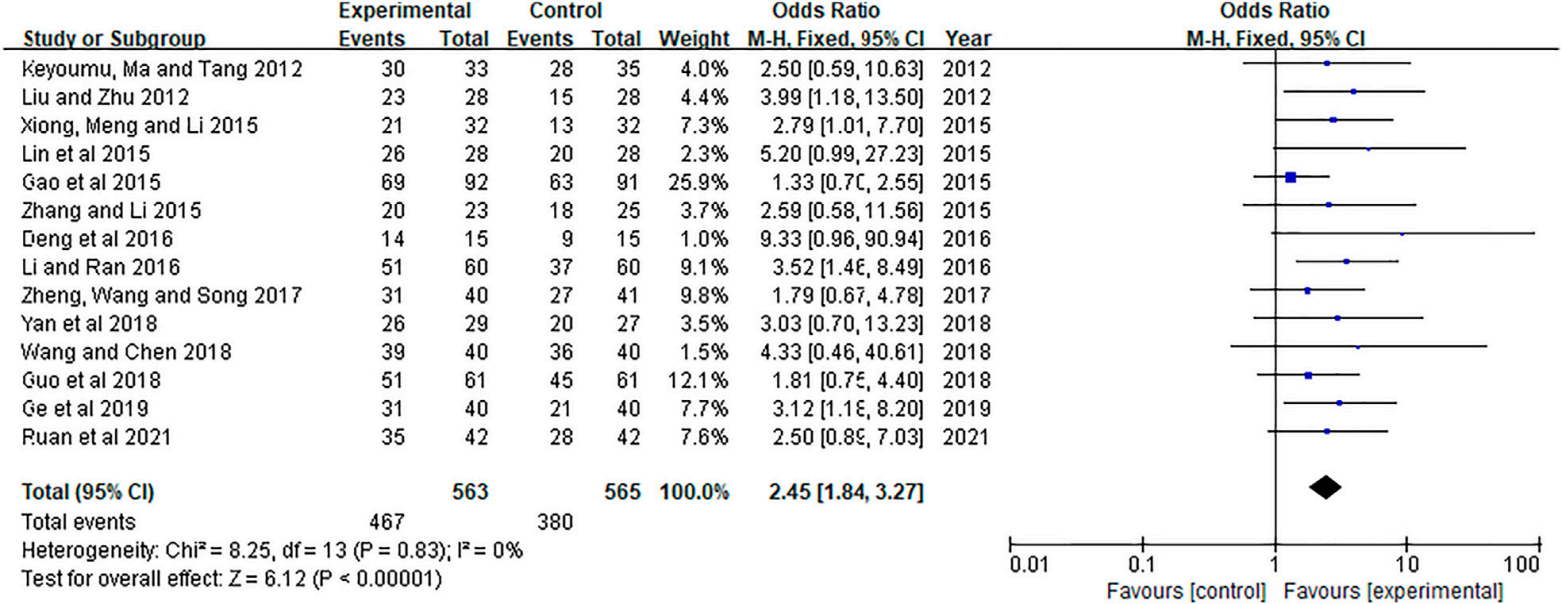
Meta-analysis of DCR.

14 literatures (Liu and Zhu 2012; Saifuding et al., 2012; Gao et al., 2015; Lin et al., 2015; Xiong et al., 2015; Zhang and Li 2015; Deng et al., 2016; Li and Ran 2016; Zheng et al., 2017b; Guo et al., 2018; Wang and Chen 2018; Yan et al., 2018; Ge et al., 2019; Ruan et al., 2021) included 1,128 patients reported DCR (DCR = CR + PR + SD). The results showed in Figure 4. The heterogeneity test showed *p* = 0.83, *I* ^*2*^ = 0.0%, so the fixed effect model was used for analysis. The meta-analysis results showed that DCR of the experimental group was significantly higher than that of the control group [OR = 2.45, 95%CI (1.84, 3.27), Z = 6.12, *p* < 0.00001].

### Comparison of KPS scores

**FIGURE 5:**
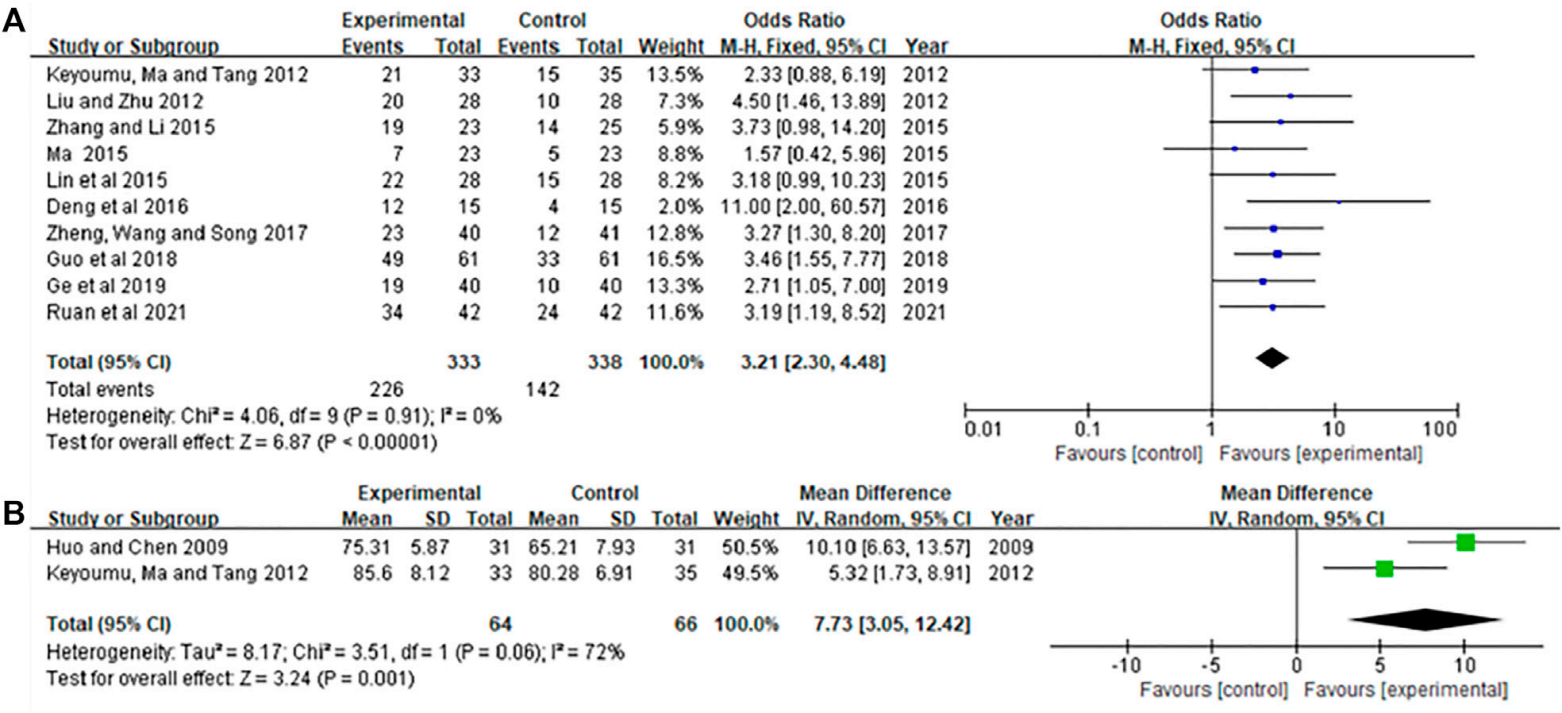
Meta-analysis of KPS. **(A)** KPS as a dichotomous variable, **(B)** KPS as a continuous variable.

In those studies (Liu and Zhu 2012; Saifuding et al., 2012; Lin et al., 2015; Ma 2015; Zhang and Li 2015; Deng et al., 2016; Zheng et al., 2017b; Guo et al., 2018; Ge et al., 2019; Ruan et al., 2021), the KPS score is expressed by the number of cases of score improvement, while less literature reports specific scores (Huo and Chen 2009; Saifuding et al., 2012). The results showed in Figure 5. The heterogeneity test in Figure 5A showed *p* = 0.91, *I* ^*2*^ = 0.0%. Therefore, the fixed-effects model was used for analysis. The meta-analysis results showed that KPS of the experimental group was significantly higher than that of the control group [OR = 3.21, 95% CI (2.30, 4.48), Z = 6.87, *p* < 0.00001]. The heterogeneity test in Figure 5B showed *p* = 0.06, *I* ^*2*^ = 72.0%. Therefore, the random effects model was used for analysis. The meta-analysis results showed that KPS of the experimental group was significantly higher than that of the control group [MD = 7.73, 95% CI (3.05, 12.42), Z = 3.24, *p*=0.001].

## Biochemical index levels

### Inflammatory factor

**FIGURE 6:**
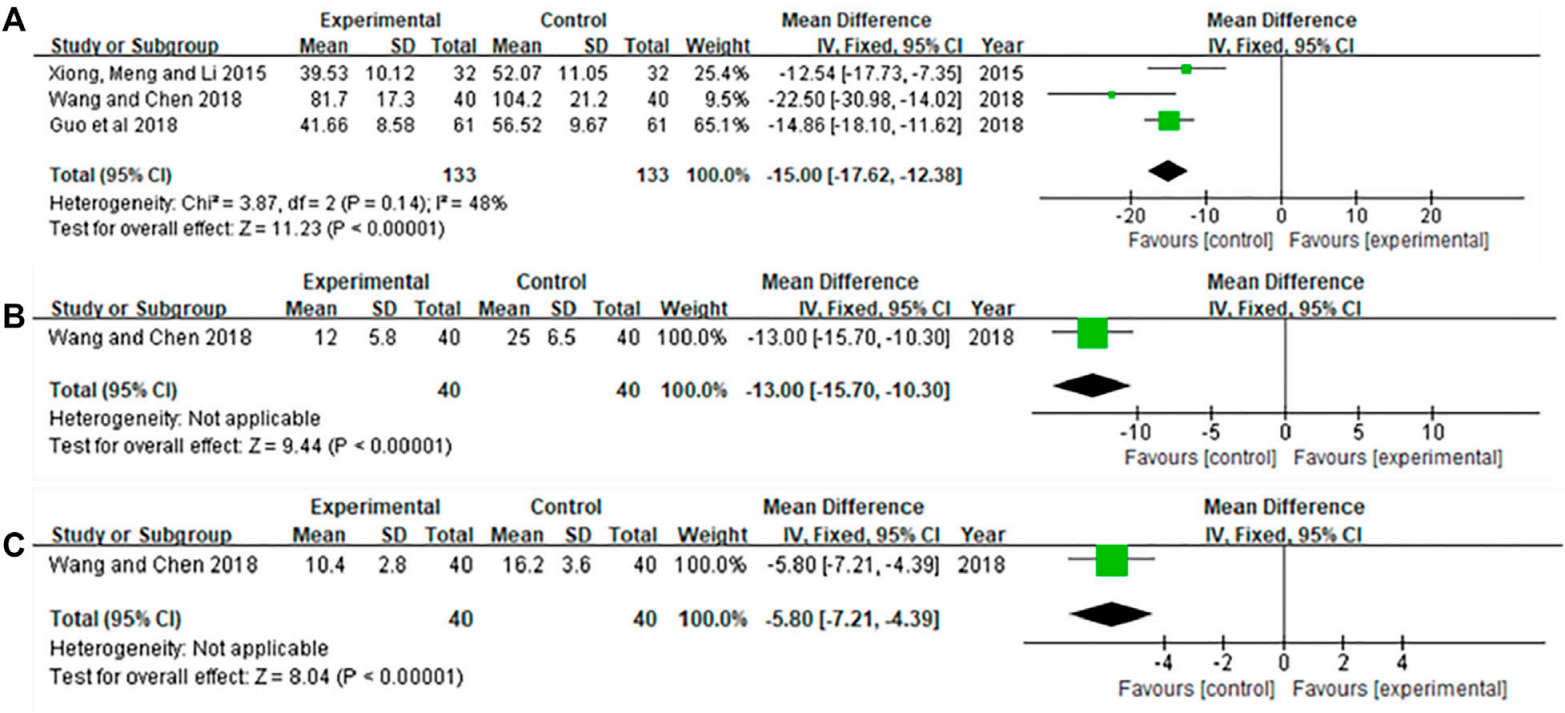
Meta-analysis of inflammation factors. **(A)** TNF-α; **(B)** IL-6; **(C)** CRP.

Three included studies (Guo et al., 2018; Wang and Chen 2018; Xiong et al., 2015) reported the level of TNF-α in serum of patients, and one literature (Wang and Chen 2018) reported the levels of serum IL-6 and CRP in patients. The results are shown in Figure 6. Figure 6A Heterogeneity test showed *p* = 0.14, *I* ^*2*^ = 48.0%. The results of the meta-analysis using the fixed-effects model and MD showed that Xiaoaiping injection could significantly reduce TNF-α levels [MD = −15.00, 95% CI (−17.62, -12.38), Z = 11.23, *p* < 0.00001]. Figures 6B,C showed that Xiaoaiping injection significantly reduced serum IL-6 [MD = −13.00, 95% CI (−15.78, -10.30), Z = 9.44, *p* < 0.00001]; CRP [MD = −5.80, 95% CI (−7.21, −4.39), Z = 8.04, *p* < 0.00001].

### Immune function

**FIGURE 7:**
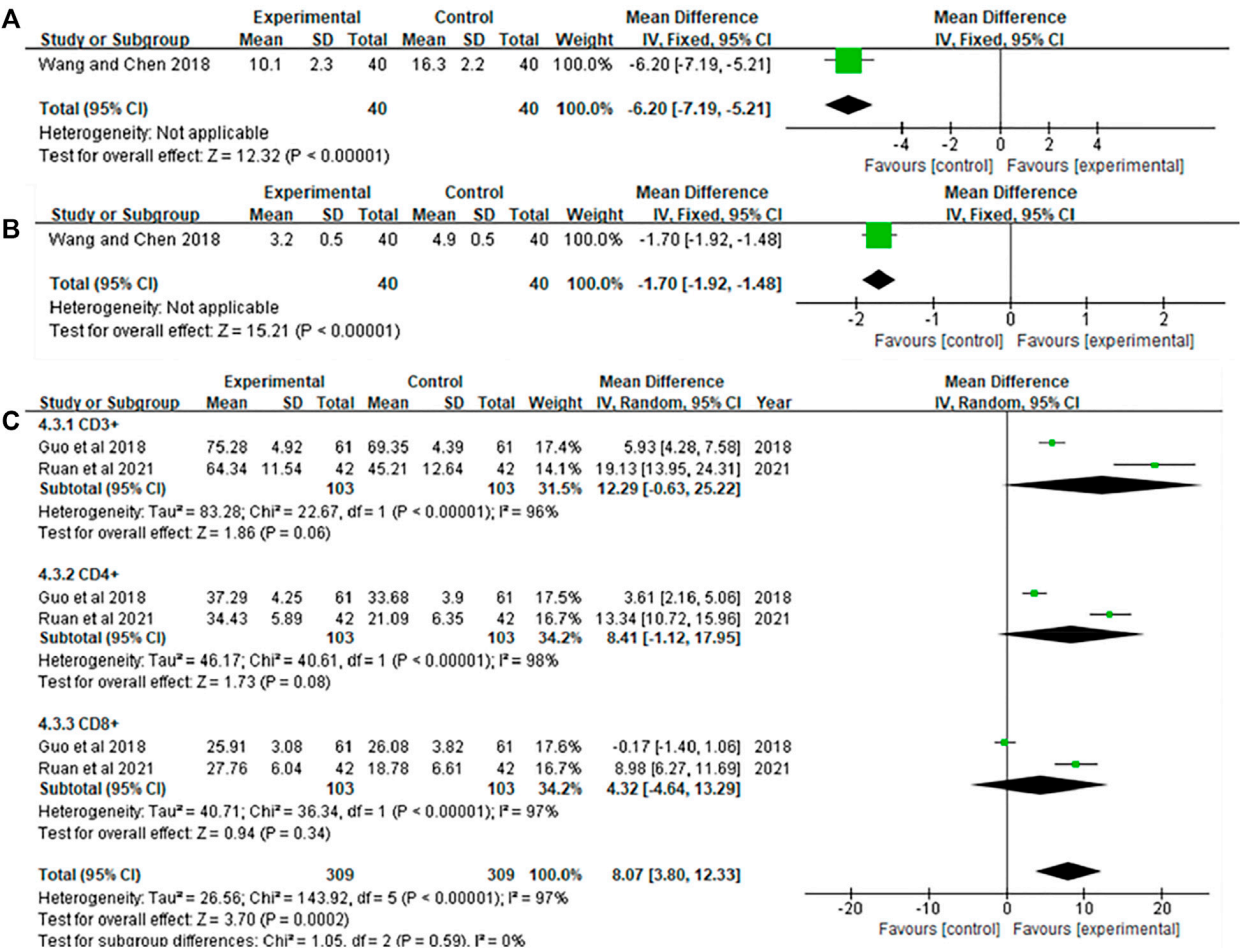
Meta-analysis of immune function. **(A)** MDSCs; **(B)** Treg; **(C)** CD3^+^, CD4^+^, and CD8^+^.

Three literatures (Guo et al., 2018; Wang and Chen 2018; Ruan et al., 2021) reported immune function, one of the articles (Wang and Chen 2018) reported the levels of MDSCs and Treg in patients, two articles (Guo et al., 2018; Ruan et al., 2021) reported patient CD3^+^, CD4^+^, CD8^+^ levels. The results are shown in Figure 7. Figures 7A,B results showed that Xiaoaiping injection significantly reduces MDSCs [MD = −6.20, 95% CI (−7.19, −5.21), Z = 12.32, *p* < 0.00001], Treg [MD = −1.70, 95% CI (−1.92, −1.48), Z = 15.21, *p* < 0.00001]. The heterogeneity test in Figure 7C suggested that the random effects model was used for analysis, and the results showed that Xiaoaiping injection combined with chemotherapy could increase the immune function of the body, but the difference was not statistically significant CD3^+^ [MD = 12.29, 95% CI (−0.63, 25.22), Z = 1.86, *p* = 0.06], CD4^+^ [MD = 8.41, 95% CI (−1.12, 17.95), Z = 1.73, *p* = 0.08], CD8^+^ [MD = 4.32, 95% CI (−4.64, 13.29), Z = 0.94, *p* = 0.34].

### Tumor markers

**FIGURE 8:**
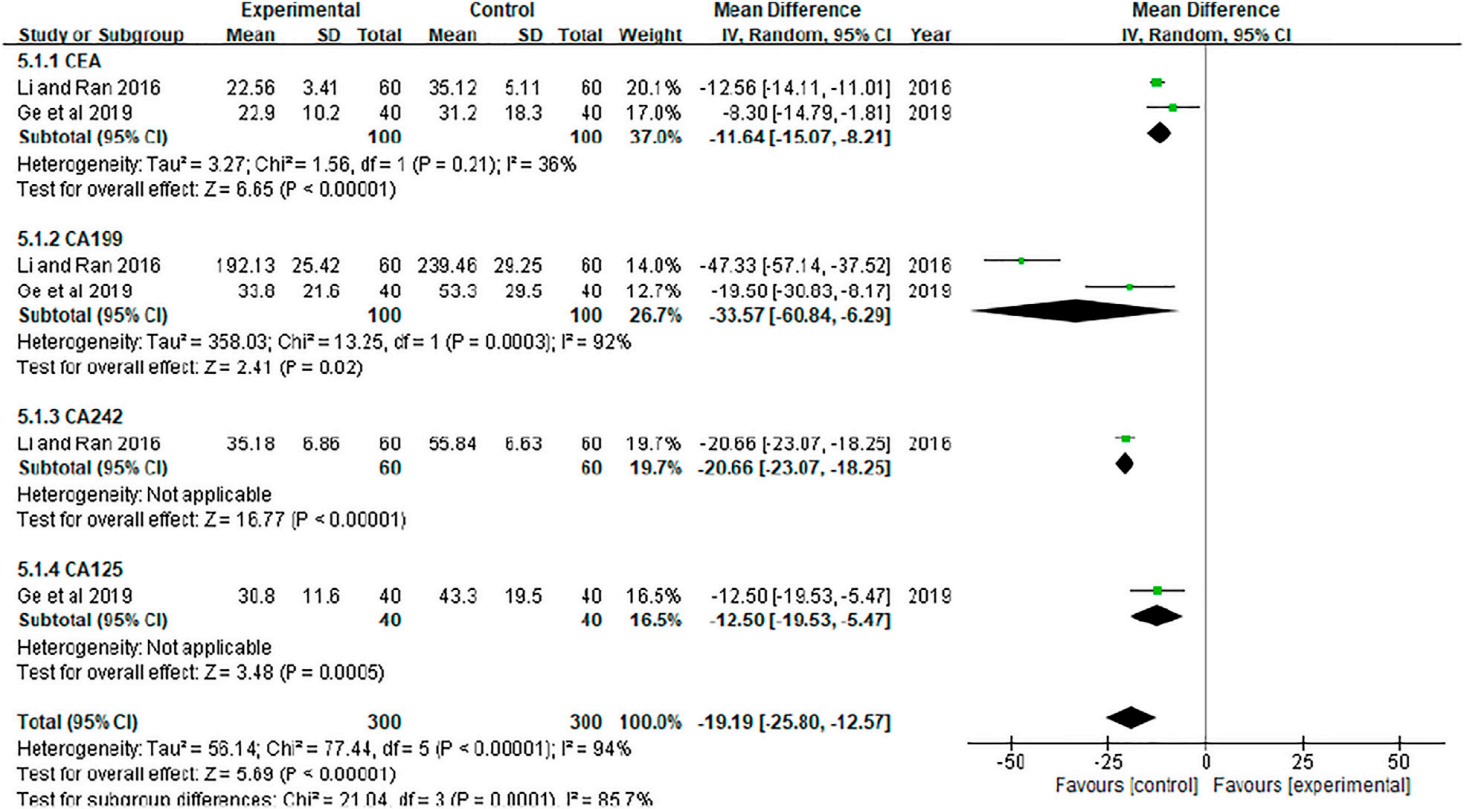
Meta-analysis of immune function.

In the included studies, two literatures (Ge et al., 2019; Li and Ran 2016) detected the levels of tumor markers in the serum of patients, of which two literatures (Ge et al., 2019; Li and Ran 2016) reported the levels of CEA and CA199, one literature (Li and Ran 2016) reported the levels of CA242, and one literature (Ge et al., 2019) reported CA125 levels. The results are shown in Figure 8. Xiaoaiping injection combined with chemotherapy could significantly reduce the levels of serum tumor markers, including CEA [MD = −11.64, 95% CI (−15.07, −8.21), Z = 6.65, *p* < 0.00001], CA199 [MD = −33.57, 95% CI (−60.84, −6.29), Z = 2.41, *p* = 0.02], CA242 [MD = −20.66, 95% CI (−23.07, −18.25), Z = −16.77, *p* < 0.00001], CA125 [MD = −12.50, 95% CI (−19.53, −5.47), Z = 3.48, *p* = 0.0005].

### Adverse reactions

**FIGURE 9:**
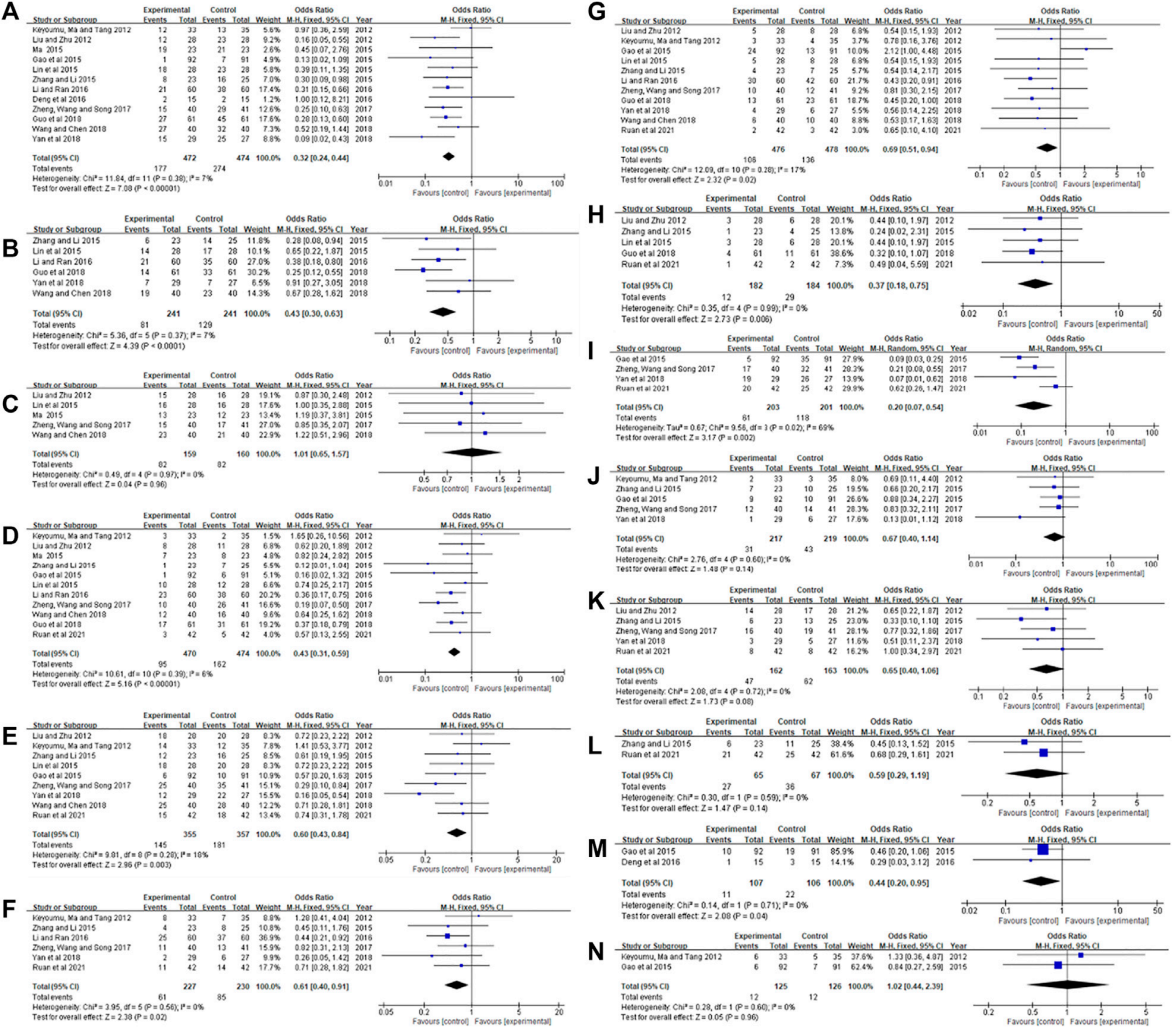
Meta-analysis of adverse reactions. **(A)** Leukopenia; **(B)** Hand-foot syndrome; **(C)** Decreased hemoglobin; **(D)** Thrombocytopenia; **(E)** Nausea and vomiting; **(F)** Oral mucositis; **(G)** Abnormal liver function; **(H)** Abnormal renal function; **(I)** Neutrophil decrease; **(J)** diarrhea; **(K)** peripheral neurotoxicity; **(L)** Anemia; **(M)** Fatigue; **(N)** paresthesia.

16 RCTs reported adverse reactions, including leukopenia, hand-foot syndrome, decreased hemoglobin, decreased platelets, nausea and vomiting, oral mucositis, abnormal liver and kidney function, peripheral neurotoxicity and other 14 adverse reactions (Figure 9). Heterogeneity test for neutropenic markers revealed *I* ^*2*^ > 50%, and random effects model was used for analysis. No obvious heterogeneity was found in the remaining 13 adverse reactions, so a fixed effect model was used for analysis. Meta-analysis results showed that Xiaoaiping injection combined with chemotherapy could reduce OR for the four adverse events, including hemoglobin [OR = 1.01, 95% CI (0.65, 1.57), *p* = 0.96], diarrhea [OR = 0.67, 95% CI (0.40, 1.14), *p* = 0.14], anemia [OR = 0.59, 95% CI (0.29, 1.19), *p* = 0.14], feeling abnormal [OR = 1.02, 95% CI (0.44, 2.39), *p* = 0.96], but the difference was not statistically significant. Xiaoaiping injection combined with chemotherapy could significantly reduce OR for the 10 adverse events, including leukopenia [OR = 0.32, 95% CI (0.24, 0.44, *p* < 0.00001], hand-foot syndrome [OR = 0.43, 95% CI (0.30, 0.63), *p* < 0.0001], thrombocytopenia [OR = 0.43, 95% CI (0.31, 0.59), *p* < 0.00001], sick and vomit [OR = 0.60, 95% CI (0.43, 0.84), *p* = 0.003], oral mucositis [OR = 0.61, 95% CI (0.40, 0.91), *p* = 0.02], abnormal liver function [OR = 0.69, 95% CI (0.51, 0.94), *p* = 0.02], abnormal kidney function [OR = 0.37, 95% CI (0.18, 0.75), *p* = 0.006], neutropenia [OR = 0.20, 95% CI (0.07, 0.54), *p* = 0.002], peripheral neurotoxicity [OR = 0.65, 95% CI (0.40, 1.06), *p* = 0.08], fatigue [OR = 0.44, 95% CI (0.20, 0.95), *p* = 0.04].

### Publication bias analysis

**FIGURE 10:**
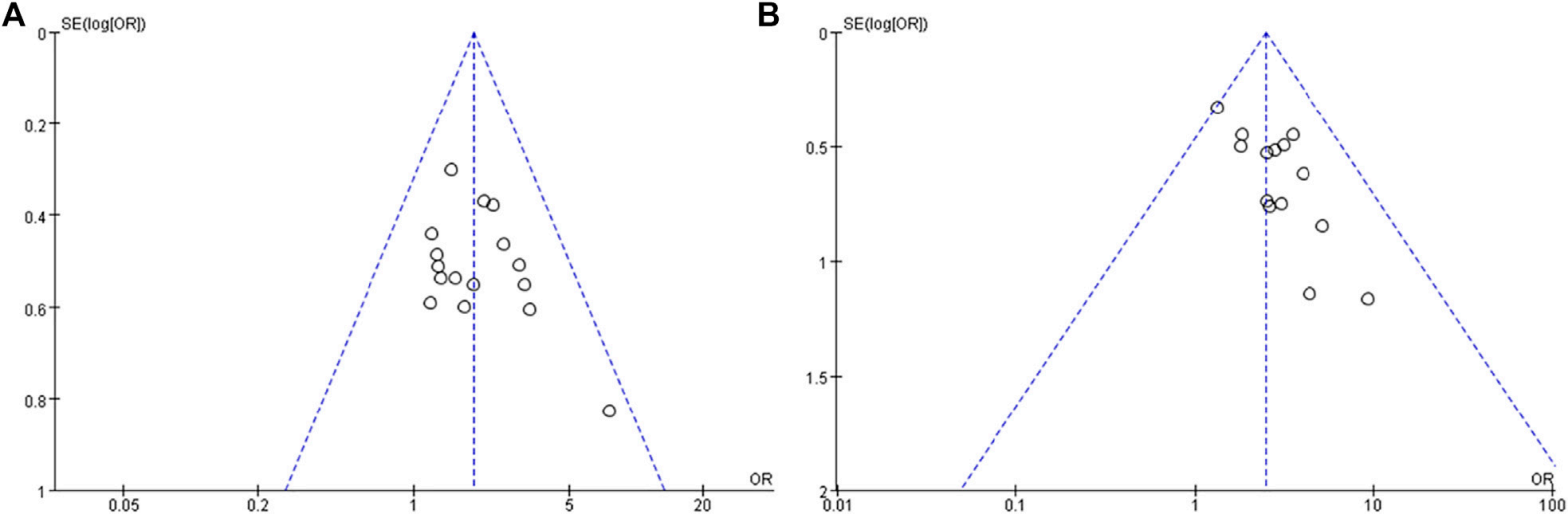
Funnel plot of the effect of RR **(A)** and DCR **(B)**.

A funnel plot was drawn according to the disease response rate and disease control rate, and the results are shown in Figure 10. It can be seen from the figure that the studies are basically symmetrical on the left and right, suggesting that the included studies can be considered to have no obvious publication bias.

## Discussion

Gastric cancer is the most common malignant tumor of the digestive system. In recent years, the detection methods have been continuously improved, but the detection of early gastric cancer is still low. It is the most commonly used method for the treatment of gastric cancer, but the patient’s immune function is affected (Xue et al., 2014). There are many adverse reactions after chemotherapy, such as leukopenia, thrombocytopenia, liver and kidney damage, oral mucositis, etc. Traditional Chinese medicine therapy has certain advantages in improving the quality of life of patients undergoing chemotherapy, and has been clinically recognized. Xiaoaiping injection is a Chinese patent medicine that completely retains the active ingredients of the medicine by adopting low-temperature extraction, bioseparation and high-tech ion exchange extraction and other modern Chinese medicine preparation processes. Xiaoaiping injection mainly included polysaccharides, C-21 steroidal saponins, organic acids and alkaloids etc., which had the effect of clearing away heat and detoxifying, resolving phlegm and softening firmness. It had pharmacological effects such as anti-tumor, antihypertensive, and antiasthmatic, and immune regulation, clinically used for gastric cancer, lung cancer, esophageal cancer, liver cancer, and other diseases (Chen and Liu 2004). Reports showed that Xiaoaiping injection, from the extract of *M. tenacissima* (Roxb.) Wight et Arn., had definite anti-tumor effects, and the mechanisms were related with promoting tumor cell apoptosis, inhibiting tumor cell proliferation and tumor blood vessel growth, and regulating immunity, etc. Li et al. (2016) found that *M. tenacissima* and its active ingredients could treat human Burkitt leukemia by inhibiting the proliferation of tumor cells and promoting cell apoptosis. Experimental study showed that the combined use of Xiaoaiping and cisplatin significantly promoted apoptosis inhibited the proliferation, migration and erosion of tumor cells, and significantly improved the anti-tumor efficacy of cisplatin (Zheng et al., 2016; Zheng et al., 2017a). Wang et al. (2010) found that *M. tenacissima* preparation (Xiaoaiping injection) could inhibit the proliferation of ovarian cancer Caoy-3 cells and arrest the cell cycle in G0/G1 phase, and its mechanism was related to the inhibition of PI3K/Akt signaling pathway. Xiaoaiping injection may also inhibit tumor development by inducing gastric cancer cells to differentiate into normal cells (Li et al., 2001). In addition, it found that Xiaoaiping injection exerts anti-tumor effect by regulating the expression of vascular endothelial growth factor receptor 2 (VEGFR2) through PI3K/Akt signaling pathway (Wang et al., 2016). It showed that Xiaoaiping could also reduce the drug resistance of tumor cells (Han et al., 2016), whose mechanism was related to down-regulation of VEGF, basic fibroblast growth factor (bFGF), etc (Ding et al., 2016). The C21 steroidal saponin Tenacissimoside A of *M. tenacissima* extract could act on HepG2/Dox tumor cells, prevent the expression of P-glycoprotein, reduce the drug resistance of tumor cells, and enhance their sensitivity to drugs (Huang et al., 2013). Other study also reported the extracts and main components regulated the immunity in order to play anti-tumor effect (Chen et al., 2010; Xing et al., 2011; Huang et al., 2013; Han et al., 2017). The results of this meta-analysis found that Xiaoaiping injection combined with chemotherapy has a good effect on advanced gastric cancer, and the improvement mechanism is related to inhibiting inflammatory response, improving immunity, and reducing the expression of tumor markers.

In the results of the quality assessment of the included literature, seven studies (Guo et al., 2018; Wang and Chen 2018; Saifuding et al., 2012; Ruan et al., 2021; Gao et al., 2015; Zheng et al., 2017b; Ge et al., 2019) mentioned use of random number tables, but nine studies (Huo and Chen 2009; Liu and Zhu 2012; Lin et al., 2015; Ma 2015; Xiong et al., 2015; Zhang and Li 2015; Deng et al., 2016; Li and Ran 2016; Yan et al., 2018) did not mention of how the random number sequence is generated. None of the 16 studies detailed the assignment method, which may lead to an increased risk of selection bias. At the same time, all studies did not blind the participants and reviewers, and were prone to subjective interference during the implementation process, and lacked the ability to evaluate the objectivity of results. The interventions included conventional chemotherapy regimens such as SOX and XELOX. The dosage of Xiaoaiping injection includes: 40 ml/d (Guo et al., 2018; Wang and Chen 2018; Zhang and Li 2015; Gao et al., 2015), 60 ml/d (Saifuding et al., 2012; Lin et al., 2015; Ma 2015; Yan et al., 2018), 80 ml/d (Liu and Zhu 2012; Xiong et al., 2015; Deng et al., 2016; Li and Ran 2016; Ge et al., 2019), 100 ml/d (Ruan et al., 2021). What’s more, the treatment time of Xiaoaiping injection combined with chemotherapy is usually 3 weeks as a course, with two consecutive courses. The dosage and intervention time of the drugs are not completely consistent, which may affect the outcome indicators. The outcome indicators of included study reported clinical efficacy and adverse reactions, and 11 literatures reported KPS score. In addition, some studies also detected the levels of serum inflammatory factors and tumor markers in patients with gastric cancer, and some studies reported the immune function of patients with gastric cancer, which were used to carry out meta-analysis. The results were basically consistent with the anti-tumor effect mechanisms of Xiaoaiping injection.

## Conclusion

Xiaoaiping injection combined with chemotherapy regimen in the treatment of advanced gastric cancer can achieve better clinical efficacy in terms of improving the effective rate and the quality of life, also reducing the incidence of adverse reactions. Since most of the studies did only observe the clinical efficacy and adverse reaction-related indicators, but not observe or report the biochemical indicators in serum or plasma. It is necessary to design the mechanism-related reports of clinical studies in the future, which will provide reference for the treatment of gastric cancer. At the same time, due to the limited literature included and the low methodological quality in this study, it is needed about more prospective, high-quality, large-sample, multi-center randomized controlled trials in the future (Hu et al., 2008).

## Data Availability

The original contributions presented in the study are included in the article/supplementary material, further inquiries can be directed to the corresponding author.

## Abbreviations

bFGF, basic fibroblast growth factor; CEA/CA, carcinoembryonic antigen; CNKI, China National Knowledge Infrastructure; CR, complete remission; CI, confidence interval; CRP, C-reaction protein; DCR, disease control rate; FOIFOX, oxaliplatin + leucovorin + 5-fluorouracil; IL-6, interleukin-6; KPS, Karnofsky performance status; MDSCs, myeloid-derived suppressor cells; MD, mean difference; OR, odds ratio; PR, partial remission; PD, progressive disease; PROSPERO, prospective register of systematic reviews; RCTs, randomized controlled trials; RR, remission rate; SOX, seggio + oxaliplatin capsules; SD, stable disease; TNF-α, tumor necrosis factor alpha; TP, paclitaxel + cisplatin; Treg, regulatory T cells; VEGFR2, vascular endothelial growth factor receptor 2; XAPI, Xiaoaiping injection; XELOX, xeloda + oxaliplatin.
